# Opportunistic bowel infection after corticosteroid dosage tapering in a stage IV lung cancer patient with tislelizumab‐related colitis

**DOI:** 10.1111/1759-7714.13401

**Published:** 2020-04-02

**Authors:** Jun Ni, Xiaotong Zhang, Li Zhang

**Affiliations:** ^1^ Department of Pulmonary and Critical Care Medicine, Peking Union Medical Hospital Chinese Academy of Medical Science & Peking Union Medical College Beijing China

**Keywords:** *Clostridium difficile*, colitis, cytomegalovirus, diarrhea, immunotherapy

## Abstract

Immune checkpoint inhibitors, the new standard in cancer therapy, present durable responses in numerous solid tumors and hematologic malignancies, as well as resulting in an increased incidence of immune‐related adverse events (irAEs). Diarrhea is a common irAE, with an incidence rate of approximately 10% to 13%. It is important to distinguish between diarrhea symptomatic of an infection, which is the main differential diagnosis, and immune‐related diarrhea. Here, we report a case of an advanced lung cancer patient who presented with diarrhea as a result of treatment with tislelizumab, a novel PD‐1 inhibitor. Although the patient initially responded to corticosteroid treatment, diarrhea recurred upon dosage tapering, and eventually improved on treatment with ganciclovir and vancomycin. Therefore, clinicians must remain highly vigilant against infection and carefully distinguish symptoms of infection from irAEs by performing repeated blood or fecal examinations for pathogens, colonoscopy, and biopsy.

## Introduction

Monoclonal antibodies targeting immune checkpoint proteins or immune checkpoint inhibitors (ICIs) have become a new standard of care for diverse cancers.^1^ Although immune‐related adverse events (irAEs) are mostly well tolerated, in some cases, they may be severe and even life‐threatening. A retrospective review^2^ reported that diarrhea, the most common irAE, occurred in 10% to 13% of patients, of whom 1%–2% discontinued immunotherapy due to intolerable diarrhea. In this report, we describe an interesting case of severe diarrhea of different etiology that occurred during immunotherapy.

## Case report

**Figure 1:**
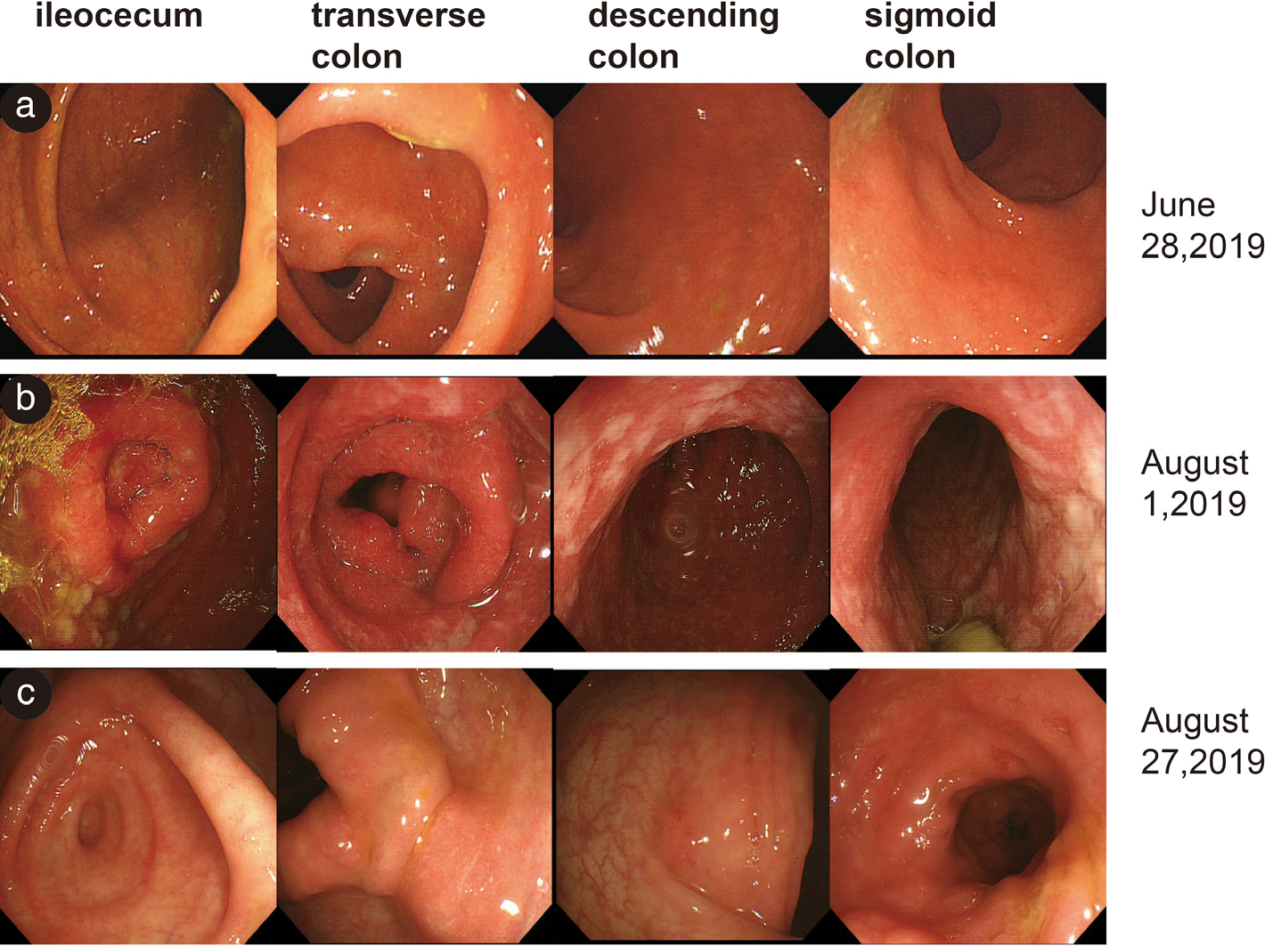
(**a**) Colonoscopy of the ascending colon, transverse colon, descending colon, and sigmoid colon showed rectal mucosal erosion, most consistent with PD‐1 related colitis. (**b**) All colorectal mucosa showed loss of normal vascular pattern, erythematous mucosa and edema, shallow erosion, mucosal crispness, and bleeding tendency. (**c**) Colorectal mucosa manifested state, with only scattered congestion in small portions of erythematous mucosa and edema.

**Figure 2:**
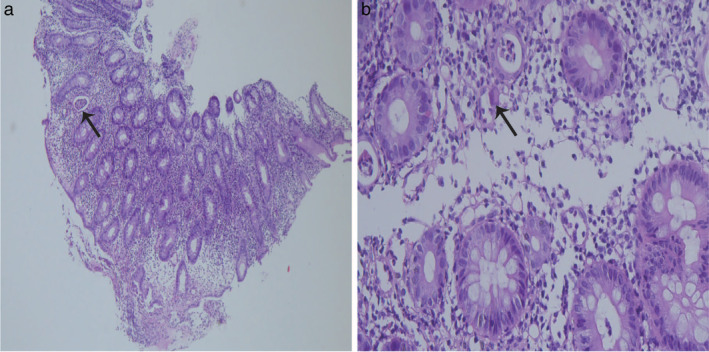
Colon biopsy following colonoscopy on 1 August 2019 showed colonic mucosa with abundant inflammatory cells and crypt abscesses (arrow in **a**) and CMV inclusion bodies (arrow in **b**). Hematoxylin and eosin stain (**a**) x40 and (**b**) x100.

**Figure 3:**
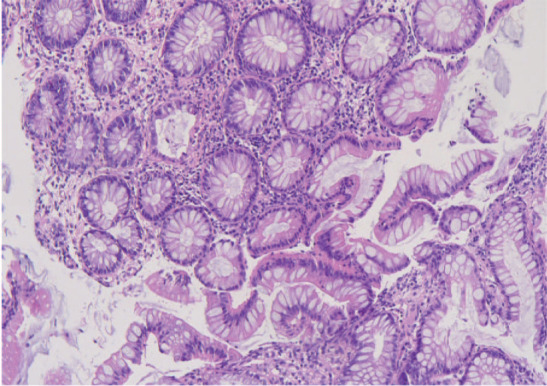
Colon biopsy following colonoscopy on 27 August 2019 showed colonic mucosa with acute and chronic inflammation. Hematoxylin and eosin stain x100.

A 73‐year‐old man with an irritating dry cough was diagnosed with cT3N3M1c, stage IVB lung adenocarcinoma, with metastases in the right supraclavicular lymph node, left scapula, and right pubic bone. Mutational analyses of ALK, EGFR, ROS1, cMET, and BRAF (V600E) were negative. The patient was recruited to the BGB‐A317 clinical trial and started on a first‐line treatment with pemetrexed (500 mg/m2, 1025 mg, per 21 days), carboplatin (AUC = 5, 600 mg, per 21 days), and a novel PD‐1 inhibitor “tislelizumab” made by a Chinese company (200 mg, per 21 days), from 28 November 2018 to 12 February 2019. The best objective response noted was stable disease. This was followed with pemetrexed and tislelizumab as maintenance therapy from 5 March 2019 to 29 May 2019. Two weeks after the last dose (mid‐June, 2019), the patient was admitted to our unit reporting watery diarrhea (8 to 10 times a day) without fever, asthenia, nausea, or vomiting. The stool assay results were negative. The abdominal computed tomography (CT) scan showed thickening of the pan‐colon wall and marked thickening of the ascending colon. Colonoscopy showed erythematous mucosa in the colon (29 June 2019; Fig 1a), and immune‐related colitis up to grade three was subsequently diagnosed. Tislelizumab treatment was stopped and supportive symptomatic treatments were prescribed. Diarrhea was alleviated by methylprednisolone administration (2 mg/kg/day; 160 mg/day) for three days without antibiotics. During the tapering of methylprednisolone dosage to 60 mg/day, the patient complained of recurrent diarrhea (4 to 5 times a day) with fever and weakness. On this occasion, cytomegalovirus (CMV) DNA polymerase chain reaction (PCR) test and the *Clostridium difficile* detection assay results were positive. The abdominal CT results were similar to the first scan results. The second colonoscopy showed nonulcerative inflammation in the entire colon mucosa (Fig 1b). Biopsy showed acute and chronic inflammation with cryptitis and crypt abscess, with occasional CMV inclusion bodies (Fig 2). Recurrent diarrhea rapidly resolved after two weeks of treatment with the antiviral drug, ganciclovir (375 mg intravenously, q.12 hours; and 500 mg capsules, t.i.d.), and the antibiotic, vancomycin (125 mg orally, q.i.d.). Meanwhile, corticosteroid dosage was rapidly decreased (by 25 mg per week), and its administration was stopped on 22 August 2019. Subsequent colonoscopy showed only scattered congestion in small portions of the mucous membrane (27 August 2019; Fig 1c). Histological examination revealed that the texture of the rectal blood vessels was blurred without CMV inclusions (Fig 3). *Clostridium difficile* assay and CMV PCR test results were negative. At the last follow‐up (25 November 2019), the patient had no diarrhea and had recovered his energy and strength. However, due to a new hepatic metastasis, the patient subsequently dropped out of the trial.

## Discussion

Immune‐related colitis is being recognized as one of the most common adverse events,^1^ with severity ranging from mild to life‐threatening. The main clinical abnormalities observed are nonspecific, including anemia, increased serum C‐reactive protein, and low serum albumin levels. While immune‐related gastrointestinal adverse events seem to arise due to severe disruption of the immune homeostasis,^3^, ^4^ their pathogenesis is not fully clear,^5^ and a predictive biomarker for routine clinical use is yet to be determined. Several biomarkers (inflammation‐induced proteins lipocalin 2, calprotectin, intestinal fatty acid binding‐proteins and tight junction proteins) present in blood, feces, or urine have been used to measure gut immune homeostasis and gut epithelial integrity.^6^ However, these biomarkers and assay are likely to be of limited clinical use.

Colonoscopy is an essential examination used to identify the cause of diarrhea. Previous retrospective studies^7^, ^8^ have reported that characteristic endoscopic findings are associated with disease outcomes in ICI‐related colitis and can thus inform treatment. These findings include exudates, granularity, loss of vascular pattern, and pancolonic ulcerations. Due to the large overlap of gross description and colitis distribution between immune‐related colitis and bowel infection, endoscopy may be unsuitable to distinguish between these conditions. Biopsy is the gold standard for diagnosis and can be used to identify the less common causes of diarrhea, such as the CMV infection in the patient reported here.

During immunotherapy, the cellular components of intestinal bacteria interact with pattern recognition receptors to promote intestinal dendritic cell activation, boost differentiation and activation of Th17 cells^9^; this is a high‐risk factor for severe gastrointestinal infections, such as *Clostridium difficile* infection (CDI).^10^ Additionally, long‐term systematic corticosteroid treatment (>30 days)^2^ and immunosuppression may be associated with an increased risk of gut infection, or virus reactivation.^11^ Corticosteroids have an immunosuppressive effect mainly by disrupting T lymphocyte and monocyte functions and blocking the production of inflammatory cytokines; thus, there is a major risk of CDI with long‐term corticosteroid administration.^12^, ^13^ Considering those factors, patients with immune‐related colitis possibly have a high risk of bowel infection.

The secondary infection was treated with ganciclovir and vancomycin. Retrospective studies^14^ have shown that the effective median course of treatment with ganciclovir is 14 days. For patients with immunodeficiency combined with CMV enteritis, the course of treatment is for 3–6 weeks, or until symptoms disappear.

As ICI‐related diarrhea can mimic the clinical manifestations of infectious diarrhea, and its treatment can itself trigger infection, the differential diagnosis of diarrhea in the context of immunotherapy needs to be done carefully. It is imperative to detect pathogens early for effective diagnosis and treatment; thus, we highly recommend that clinicians should rule out infectious colitis when diagnosing patients with immune‐related diarrhea.

## Disclosure

The authors have no potential conflict of interest to disclose.
